# Of Heart-Clot as a Cause of Death in Diphtheria

**Published:** 1864-06

**Authors:** J. Forsyth Meigs

**Affiliations:** Fellow of the College of Physicians of Philadelphia, one of the Physicians to the Pennsylvania Hospital


### Selections.

### OF HEART-CLOT AS A CAUSE OF DEATH IN DIPHTHERIA.

By J. FORSYTH MEIGS, M.D., Fellow of the College of Physicians of Philadelphia, one of the Physicians to the Pennsylvania Hospital.

Reprinted from the "American Journal of the Medical Sciences."

It is now somewhat less than four years since my attention was directed to the formation of coagula in the cavities of the heart, as a cause of death in certain cases of diphtheria.

The *first case* in which I observed this accident, or result, occurred in my own practice, in November, 1860, in a little girl, between six and seven years old, healthy in every respect, and born of vigorous parents. She was one of four children, all living at the time, and this case was the only one which then occurred in the family. The attack was severe, the deposit covering the whole of both tonsils, the uvula, and a considerable portion of the posterior surface of the pharynx. The external lymphatic ganglions were very considerably implicated, the swelling and soreness being marked, but there was no œdema under the chin. The constitutional symptoms were severe, but not malignant, the appetite, in particular, being almost null. The treatment consisted of stimulants and nutrients internally, and of cayenne pepper infusion applied to the fauces, and a stimulant embrocation used externally. At the end of the second week, the disease had seemingly yielded, and, on the nineteenth day, the local symptoms had disappeared. The strength was so much improved that the child sat up in bed from time to time stringing beads, and, in the morning of the day named, she was placed in a large arm-chair for an hour. This was the first occasion on which she had been allowed to leave the bed after the onset.

On the afternoon of that day (the nineteenth) the patient was not so well. She was paler than she had been for some days, seemed weaker and more listless, and was more restless the night following than for some nights before. On the following day, there was again the same listless appearance about the child—a weariness, inattention, paleness of the face, a weakly pulse, poor appetite, but nothing more express. Towards evening, I was still more struck with this appearance of general and inexplicable debility. And yet the muscular power was not seemingly much reduced, for, though there was no desire to sit up, the child moved freely in bed, turned, rose up and lay down without any difficulty whatever. The pulse, though feeble, was regular, between 90 and 100; the respiration easy, and no abnormal sounds to be heard either in the lungs or heart.

On the following day (the twenty-first) in the morning, I was, for the first time, seriously alarmed. The general expression was most curiously that of intense weariness, of extreme lassitude, without the slightest appearance of pain or distress anywhere. The circulation, however, was evidently failing. The pulse was still not over 100, its rhythm was correct, but the force of the arterial pulsations had lessened and become somewhat irregular, so that they were smaller and feebler than natural, and some much feebler than others. The temperature of the body was normal. The color of the surface was paler and whiter than usual, but there was no cyanotic tint of any part. The muscular movements, as performed in bed, were natural, quick, and sometimes petulant. The patient could lie in any position, used but one pillow, had no disposition to be raised up, and gave no sign whatever of even a tendency to dyspnœa. The mind and senses were as bright as ever, and the temper, beyond occasional slight fretfulness, was good. There was no appetite, but she took milk punch and stimulants when desired.

I now examined the lungs most carefully. There was nothing abnormal about them. Then turning to the heart, which I now felt must be the seat of trouble, I sought for endocarditis or pericarditis, but, in the absence of distinct murmur or friction, of increase in the area of dulness, or of altered position of the apex-beat, and, determined too by the entire absence of fever or pain, I was satisfied that there was no acute inflammatory condition to explain the state of the circulation. What remained? The formation by slow, but continuous, concretions, within the cavities of the organ, of a mechanical impediment in the systemic circulation, the external signs of which were to be seen in the gradual, but relentless, failure of the child's vitality, and especially in the weakening pulse, and the pale surface, whilst there was not a sign of cerebral, pulmonic or digestive disorder of a serious kind. Though I have said there was no murmur nor friction in the heart-sounds, there was a curious deviation from the natural condition, which it is difficult to describe. The sounds were confused, indistinct, and seemed as though reduplicated.

From this time the patient grew gradually worse. Stimulants with bark were freely administered, but to no purpose. The pulse became smaller and more feeble, and irregular in force, though not in rhythm, until finally it was a mere thread. The general pallor became deeper, but without cyanotic hue. The face was somewhat flushed, but, in other respects, natural. There was no trace of dyspnœa, no pain, no clouding of the mind, no cough, no febrile heat, and, a few moments before the child died, she spoke out in clear, distinct tones, and turned without any difficulty in bed, suddenly, and with force. The death was sudden, as in other cardiac diseases, about eight o'clock in the evening of the twenty-first day.

I regret that no examination of the urine was made either before or after death. At that time my attention had not been drawn so decidedly as now to the condition of the renal secretion.

*Autopsy.*—The lungs and digestive organs presented nothing abnormal. The pericardium was quite natural. The heart appeared somewhat larger than usual, owing to the fact that the right auricle was distended with a dark, jelly-like mass of coagulated blood. A large coagulum was found also in the right ventricle; this was firm, yellowish-white in color, adherent to the interior structures of the cavity, and, from its appearance, had evidently been some time in forming. A smaller coagulum was found in the left ventricle.

Of the *second case* I have but few notes, and therefore its history will be brief and imperfect. The subject was a girl between 7 and 8 years old, of good health. She was one of quite a large family, in which the disease was epidemic, the fatal case having been preceded by a very severe one, which, however, ended favorably, in a younger child, and being followed by another fatal case from croup, and by a less severe one in an older child.

This case began January 2d, 1862. It was severe from the onset, the throat affection being marked by extensive exudation, and by great tumefaction both interiorly and exteriorly. The case continued to present the usual symptoms up to the third week, when the local symptoms gave way, and I supposed that the attack was about to terminate favorably; but the child, instead of improving decisively, continued weak and languid. The circulation became feeble, as marked by general pallor of the skin, a small but not very frequent pulse, growing day by day more and more feeble, and a slowly increasing general debility, without any disturbance of the cerebral or general nervous system, or of the respiratory or digestive functions. In this case, also, by the method of exclusion, I formed the opinion that the cause of the gradually increasing vital exhaustion lay in the circulation, and that it must be the result of coagulation of blood in the cavities of the heart. The child died on the 25th day from the onset, without any respiratory struggle, without pain, without marked or painful phenomena of any kind, with simply the signs of a slow, but constantly progressive impediment to the circulation.

The treatment consisted, as in the other case, of rest in bed from the first; capsicum gargle internally, and a stimulating embrocation to the outside of the throat; small doses (1-12 gr.) of Kermes mineral with Dover's powder on the first few days, and afterwards doses of iron, and milk punch or wine whey, with animal broth from the onset.

I was assisted at the *autopsy* by Dr. Packard, of this city. The lungs were healthy in all respects, as were also the digestive organs. The pericardium showed nothing abnormal. On opening the heart, we found coagula in the cavities of both sides. These coagula were large, firm, of a dark tint generally, but dotted through with yellowish-white points. They were so firm as to force the conviction that they had been formed for a number of days. In the left ventricle, the coagulum, which filled the cavity of the organ very completely, presented some singular characters. Its lower extremity, that which corresponded to the apex of the ventricle, exhibited a broken, irregular, uneven, and frayed or granulated appearance, so as to suggest at once to our minds the idea of its having been whipped and crumbled away in minute granular portions under the crushing influence of the ventricular contractions. We could not but ask ourselves whether this was not a case of heart-clot in which there was an evident attempt of nature at cure; the mode of cure being a process of slow disintegration of the coagulum from causes acting within the clot, whilst the minute, granular, almost molecular portions thus loosened, were detached by the churning action of the ventricle, and conveyed away to distant portions of the system, there to be arrested, and then finally absorbed under the marvellous selective power of the absorbent system of the body.

This was the only case of the three that I relate, in which conditions of the clot were present, exhibiting the method by which nature strives to remove from the heart the dangerous mechanical impediment to the circulation.

The *third case* occurred in F. L., a girl, aged seven years, of healthy constitution, of unusual intelligence, and most active in all her habits. She was seized with what, for a few days, left me in doubt whether the attack was to be one of true diphtheria, or merely ordinary tonsillitis, with a slight accidental exudation of false membrane in small dotted points. The general symptoms were very mild at first. In the second week the increase in the amount of the exudation, the considerable tumefaction and soreness of the cervical lymphatic ganglions, the loss of appetite, the continued febrile movement, and the decided, though moderate general weakness, showed very plainly that the case was one of diphtheria. The appetite now flagged very much, deglutition became painful, and the temper grew irritable and peevish, so that it was very difficult to nourish the patient sufficiently. At the end of the third week the throat was better; the exudation, which had never extended beyond the two tonsils and the velum, was diminishing in extent, and the external swelling had almost disappeared. The pulse was between 90 and 100; the skin only at times rather warmer than usual, the muscular strength not greatly reduced, since the child could move freely in her bed, rise up and lie down, and sit up for some length of time to play with her toys. The appetite was poor, but by urgent persuasion the child was made to take a sufficient amount of food daily, in the form of milk punch, wine whey, animal broth, milk toast, tender meat, or some such articles of diet.

At the risk of being tedious, I must state that, though at this time the local symptoms were favorable, and the general condition, as shown by the pulse, respiration, digestion, and the state of the circulation, not positively discouraging, I felt uneasy and anxious about the prospects of my patient. The child made no decided progress towards full recovery. She was listless; the pulse was rather feeble; the surface pallid, and she had a peculiar weary expression of countenance, so that, warned by past experience, I began to fear the existence of, or a tendency to, the same accident I had already witnessed twice before, the formation of cardiac coagulum.

The urine, in its general appearances, had been natural, and was so even now, except that the whole amount was rather less than usual. Upon exposing it to heat, a very moderate amount of albumen in a granular form appeared, and thickened the fluid.

About this time (21st day), the mother left the child's room for a few hours to get some exercise in the open air, and, on returning, was shocked and distressed at the extremely pale, white, and weary look of the child. So strong was the impression that she declared nothing should induce her to leave the house again until the degree of danger was definitely determined. From this time the patient was not so well. The symptoms were peculiar, and unlike, in many respects, those of any morbid condition that I am in the habit of meeting with. The intelligence and all the senses were perfect in their integrity. The mind was as bright as in the best health, and, except that the temper was rather quick and irritable, and somewhat odd, the emotional nature was healthy. The muscular strength gave way very slowly. The patient could, at all times, move the head and arms freely, and, up to three days before death, could still rise up in bed. In the last few days she complained of weakness in, and difficulty of moving the legs —evidently a state of partial, and only partial paralysis. There was nothing like paralysis of the pharynx; and the voice, after being somewhat husky, had become clear again. The lymphatic swelling had mostly disappeared, and the fauces had almost regained their natural condition. The respiration was perfectly easy, not hurried, and there was no cough, except occasionally a loose laryngeal cough, which had replaced the previous stage of husky voice.

There was no serious dyspnœa, as the child could lie with the head and shoulders low, but the respiration was sometimes suspirious. This latter condition increased during the last two days, and especially during the last twenty hours, so that long-drawn and plaintiff sighs were more and more frequently heard, something like those which occur in the early stage of tubercular meningitis, and which told the practised ear that one of the great centres of vitality was seriously deranged. The circulation failed gradually. Each day there was a slight falling off in the power of this great system. The surface grew paler and somewhat ashy, but *not* cyanotic; it had a shrunken look. The face looked thin and anxious, or, rather, weary. The expression was peculiar. It was not what we expect to see in death from embarrassed circulation. There was no lividity, no frightened and staring eye, no pleading look, as in cardiac asthma or orthopnœa, but simply an air of intense weariness and fatigue, like that of one utterly fagged out with a long and hopeless contest with a deadly enemy. This, as well as I can describe it, was the character of the facies in this case, and in the other two which I have watched. In the absence of more specific and positive symptoms of this condition, I take the above mentioned peculiarities of the physiognomonical expression to be of considerable importance.

The pulse was feeble and small during the last week of life, these conditions becoming more and more marked as time went on. It was not frequent, running usually from 80 to 100. It was irregular in force, towards the last intermittent, but not very irregular in rhythm. During the last day it was very small, weak, thready, and with considerable intermissions. There was nothing particularly to be noticed about the heart-sounds—indeed, the child was so fretful, when disturbed, that no proper examination was made in the latter part of the attack.

The urine continued moderate in quantity, rather dark in color, without deposit, and with a notable, but not large precipitate of albumen on boiling.

During the last week, I had stated my fear that the cause of the constant declension in the patient's state was the gradual formation of a coagulum in the heart, this being slowly added to and increased in size by daily concretions forming upon its exterior surface. I was assisted during the last week by one of our best practitioners, and during the last two days by a third gentleman, to both of whom I expressed freely my opinion. They regarded the case as a singular one, but were not as well satisfied as myself in regard to the immediate cause of the constantly failing circulation.

As death approached, late in the evening of the 28th day, the general debility became more and more marked, so that the child would ask to be assisted in changing her position. The mind and senses continued quite clear; the respiration, saving its sighing character and occasional suspensions, was not visibly difficult nor labored; the swallowing was not materially affected except from weakness; the pulse became a mere thread, and, for half an hour before death, could scarcely be felt; the extremities became cool and then cold; the child was drowsy and inattentive; there came a longer suspension of the breathing, a slight general convulsion, repeated two or three times within a space of fifteen minutes, and the young life had gone out, under some strange cause of embarrassed circulation, which, for a whole week, had been as tenaciously and as gradually blocking the general circulation as the slow and yet continuous formation of false membrane in the larynx, in true croup, blocks and finally arrests the function of respiration.

The treatment of this case was as follows: The child was confined to a bed or sofa from the start, being allowed to sit up to use her toys, but not permitted to run about the room. For the first few days chlorate of potash, in doses of five grains every two hours, was administered. After this, one grain of sulphate of quinia in solution with five drops of the tincture of the chloride of iron, were given every three hours, until a few days before death, when the child would no longer take it. She was then ordered a teaspoonful of elixir of cinchona, every two or three hours. When the urine was found to be scanty and albuminous, a mixture of acetate of potash, spirits of juniper, and spirits of nitre was given alternately with the iron and quinia. This soon sickened the stomach, when the spirits of nitre was given alone in water.

Throughout the case the diet was nutritious, consisting of milk-food, with bread and animal broths, and tender meat, when the child would take it. Early in the case brandy was added to the milk, and, as it progressed, the quantity was increased, or it was changed to wine whey, as it was found possible to induce the patient to use them.

The local treatment consisted at first in the use of capsicum infusion as a wash internally, with an embrocation of tincture of cantharides and spirits of turpentine applied externally from time to time. Later in the disease, alum in powder, mixed with sugar, was applied to the fauces.

*Autopsy.*—Lungs natural, except that they were paler and more crepitant than usual; they exhibited no signs of collapse, and only very slight appearances of congestion at the bases behind.

Pericardium natural; no effusion beyond a few drachms, and no trace of unusual vascularity, or of exudation. The whole size of the heart rather greater than usual, and the right auricle very much larger than the left (four or five times); blackish in color, and much distended. On opening the cavities, no trace of inflammatory action. The right side of the heart presented a firm, dark-colored coagulum, filling up the right auricle, and exhibiting prolongations into both cavæ. These prolongations were dark-colored in their general aspect, but here and there showed oblong points of a yellowish-white color. They were firm enough to retain their shape on handling, and to bear some traction before they broke. The coagulum in the auricle was continued through the tricuspid orifice into the right ventricle, filling up to all appearances, almost completely, the orifice. In the ventricle it occupied a large part of the cavity of that chamber, was adherent closely to the curtains of the tricuspid valve, to the chordæ tendineæ, and to the columnæ carneæ. It was here much lighter in color than in the auricle, being whitish or yellowish-white in its tint, and having also a much firmer and more solid consistence. In addition to the large coagulum occupying the cavity of the ventricle, there were thin layers of fibrinoid-looking concretions lying in amongst the columnæ carneæ, and resting directly upon the lining membrane of the ventricle. These were adherent with a very considerable force, but could be stripped off. They reminded me, except that they were dark in color from the coloring matter of the blood, of the exudation I have so often peeled off from the laryngeal mucous membrane. A large, firm, and dark-colored cylindrical coagulum extended into the pulmonary artery, and was traced beyond the point where the vessel bifurcates.

The left auricle was small, contracted, and contained only a very small, dark-colored clot. The left ventricle was of natural size, and contained a rather firm, slightly adherent, dark-colored coagulum, which sent a small prolongation into the aorta.

*Remarks.*—I am well aware that the three cases given above are but imperfectly reported. Any one, however, who has been immersed, body and soul, for a series of years in a large private practice in an American city, will know how to make allowance for such imperfection. Even with these imperfections, I believe them to be important and worthy of publication, since they call the attention of the profession to a mode, if not a direct cause, of death in diphtheria, which thus far has not been, I believe, recognized and described. Certainly the above three cases, occurring in my own practice within a period of four years, show that this accident or condition cannot be very rare. During this period, though I have attended a great many other cases of the disease, most of them of a mild type, I have had only three other fatal cases, and all of these died from the extension of the exudation into the larynx. In neither did I suppose that heart-clot had formed, and in neither was an autopsy made. I have seen, besides these three cases in my own practice, four fatal cases in consultation, all of which died early in the disease with acute malignant symptoms.

A peculiar interest attends such cases as those above described. The patient may or may not have presented alarming symptoms at first, but these have in a great measure disappeared, and the anxiety of the family and physician, as the local and general symptoms subside, may, and indeed must, have greatly lessened, when, suddenly, or very gradually, the case assumes a new aspect. Without any very severe or threatening symptoms, the experienced physician *feels* that some new danger approaches. The sudden arrest in the progress towards health; the continuance of certain general symptoms, such as weakness and altered temper, in spite of the improvement in the local signs; the pale face and weak pulse, and the air of general and inexplicable lassitude, beget a vague anxiety which hardly takes shape in time to save the physician the mortification and distress, and the family the anguish, of a sudden and unexpected catastrophe. I have seen, both in our own and in foreign journals, accounts of cases in which sudden and unexpected death has taken place during an apparent convalescence, in which the death has been supposed to be the result of exhaustion or syncope, or in which no attempt has been made to explain the cause of the sudden fatality. From the character of the symptoms detailed in some of these instances, I have no doubt that they were examples of the kind I have described above.

As to the cause of the coagulations I have described, I have only a few remarks to make. In 1849, my father, Dr. Charles D. Meigs, called attention to the accidental formation of heart-clots in parturient women, as a result of syncopal conditions, occurring in subjects who had already lost blood by hemorrhage during or after labor. He supposes that loss of blood increases the coagulability of what remains in the system, and that the syncopal state, occurring in women who have undergone hemorrhages, invites or conduces coagulation in the heart, through the slow and partially suspended movements of the heart during the existence of the fainting condition. He reports one case (*Obstetrics: the Science and the Art*, 4th ed., Philada., p. 339) in which a heart-clot seems to have formed on the day after the labor, and in which the patient lived to the eighteenth or nineteenth day. At the autopsy "a white, fibrinous coagulum was found in the right auricle, nearly filling it, and projecting through the tricuspid valve into the right ventricle; the tail of the clot was whipped into cords by the threshing action of the chordæ tendineæ of the ventricle. The pleura of the right cavity contained a large quantity of serum." If, however, the syncopal condition is, in truth, in this class of cases, and in others that I have met with, the real exciting cause of coagulation in the cardiac cavities, as I fully believe, this explanation will not apply to the production of coagula in diphtheria.

I shall merely suggest a mode of explanation which has occurred to me. This is that some peculiar change takes place in the constitution of the fluids or tissues, more or less akin to that which gives rise to the exudation of the diphtheric deposit on the mucous surfaces, which does, in certain instances, by an analogous power or action, induce the formation of coagula upon the interior structures of the cardiac cavities. In the *London Lancet* (vol. II., 1863, Nos. VI. and VII.) are two Croonian Lectures, on the Coagulation of the Blood, by Mr. Lister, in which the writer supposes he has shown that the tissues, when deprived of their vital properties, comport themselves like ordinary solids, and are capable, by a kind of catalytic action, of inducing coagulation of the blood which comes into contact with them. He says:—

"Thus, when an artery or vein is inflamed, coagulation occurs upon its interior in spite of the current of blood, precisely as would take place if it had been artificially deprived of its vital properties."

If this theory be borne out by further observation, it would be necessary to look with great care, hereafter, to ascertain whether the cardiac coagulum of diphtheria has been preceded, or is accompanied, by the evidence of endocarditis. In the cases reported above, I did not find any of the usual appearances of endocarditis.

Can they be prevented? Entirely ignorant as we are in regard to the particular variety of the disease, in which we should anticipate their formation, we can have no better rule of treatment than to act in such a way as to get rid of the disease as rapidly and thoroughly as possible; or, in other words, to make assiduous use in all cases, mild and severe, from the very outset of the malady, of the remedies and various hygienic measures which observation and experience have shown to be most powerful in combating this dangerous blood disease.

Does the patient ever recover after a coagulum has formed in the heart? I am disposed myself to hope that, in some rare instances, and under very favorable circumstances, nature may be able to rescue the patient from even the abyss of danger. There is no doubt that subjects recover after the formation of thrombi in the vessels, and I believe it is generally acknowledged that some instances of recovery have takan place after the discharge of emboli through the great vessels of the heart, these emboli having been either carried through the heart from some point in the venous system where they had been formed, or having been formed originally in the cavities of the heart. M. Virchow supposes that the coagula or thrombi formed in the vessels undergo a softening process from the centre outwards, which reduces them to a puriform substance, "a puriform but not a purulent substance" (*Cellular Pathology*, Am. ed., p.

34). He does not describe any such softening process in coagola located in the heart, but infers that such disintegration may take place in large fragments of thrombi lodged in arteries. Referring to thrombi carried through the right side of the heart, he says, page 241:—

"I believe, namely, that when a considerable fragment of a thrombus becomes wedged at a certain point in an artery, it may in its turn crumble away through the onward pressure of the blood, and thus the minute particles to which this crumbling of the larger plug gives rise be conveyed into the small branches into which the vessel breaks up. Thus alone does it seem to me that the fact can be explained, that in the district supplied by an artery of considerable size a number of little deposits of the same sort are often found."

In one of the cases reported by me, the second, an effort was clearly being made by nature to break up and wash out from the left ventricle the clot which had there formed. Whether such an effort can ever be successful, is the question now under consideration. It cannot be answered with certainty. To know that the effort was being made, is at least some encouragement. Any one who will read the description given above, from M. Virchow, of the conditions observed by him in large fragments of thrombi carried into the larger branches of the pulmonary artery, cannot fail to see how closely they resemble those observed by myself in the case just referred to. I may mention that this case was observed before the publication of M. Virchow's work in this country, and that I could not, therefore, have been led to look for the changes described by the expectation aroused by the perusal of M. Virchow's facts and opinions.

Whether the metastatic inflammations and suppurations which M. Virchow seems to suppose always ensue, as a consequence of the conveyance of the particles or fragments of the disintegrated thrombus or clot into the vessels beyond the point of formation of that thrombus or clot, must necessarily follow, is a question which I do not believe is yet positively determined. I think it may fairly be questioned whether the arrested fragments or molecules of the disintegaated coagulum, or thrombus, may not be absorbed, after undergoing some preparatory transformation like the fatty degeneration of inspissated pus, described by M. Virchow, without the invariable and necessary inflammatory changes supposed to constitute the basis of the metastatic conditions.

Several years ago I saw a case in consultation which, at the time, was extremely puzzling to me. It was that of a boy six years old, remarkably stout in build, hardy in constitution, and born of parents, on both sides, of the most vigorous and robust health. He had that characteristic which is so important in severe illness, toughness, tenacity of life, which makes the resurrections occasionally met with by all medical men in the course of a long experience. This boy had had a severe attack of scarlet fever, and had passed safely through the eruptive stage. During the desquamation he was attacked with albuminuria, and, when I saw him, he was passing a very scanty amount of urine per day, which was highly charged with albumen, and which the microscope showed to contain blood-globules, very numerous and large fibrinous tube-casts, and much exfoliated epithelium. He had extensive anasarca, was much exhausted, and continued very ill for many weeks. He became emaciated, very pallid; and, after the dropsy had disappeared, and the urine had greatly improved in quantity and quality, had the following curious train of symptoms. He was very feeble, so that he made no attempt to leave the bed, but he had no cerebral disorder, no material disturbance of the respiratory organs, and was always able to take his stimulants and nutrients, which were administered in large quantities, and which, with iron, constituted the essential treatment. His liver, however, became enormously enlarged, so that it extended down somewhat below the umbilicus, and then around to the crista of the right ilium. It was not painful to the touch, nor did he complain of any spontaneous pain in that region. At this time the heart-sounds had the same curious characters that were noted in the first case described above; they were indistinct, and gave at the same time the impression that they were reduplicated. The impulse was distinct, but feeble. The child continued for many weeks in this state, exhausted, emaciated, prostrate, and then began very slowly to mend; and finally, after having been in the very jaws of death, recovered entirely. Since meeting with the cases detailed in this paper, I have thought it not unlikely that the boy just alluded to may have had, and may have survived a heart-clot. His health was something quite unusual, both in fact, and by the law of descent, and I do not know that a recovery from a heart-clot, by the process above hinted at, would be more extraordinary than one from a wound, with loss of brain substance, caused by the passage of a tamping-room through the anterior portion of the cerebrum, or that of the Count d'Aumale, described by Parè, who recovered after a spear had been driven from front to rear, through the upper regions of the brain.

